# P-198. Development and Implementation of a Research Nurse Training Curriculum at the Center for Human Development in Rural Southwest Guatemala

**DOI:** 10.1093/ofid/ofaf695.420

**Published:** 2026-01-11

**Authors:** diva M barrientos, Neudy C Rojop, Eduardo M Barrios, Andrea Chacon, Kareen Arias, Alejandra Paniagua-Avila, antonio Bolaños, Claire Bradley, Edwin J Asturias, Dan Olson

**Affiliations:** Fundacion para la Salud Integral de Los Guatemaltecos, Fraijanes, Guatemala, Guatemala; Fundacion Para La Salud Integral de los Guatemaltecos, Los Encuentros, Retalhuleu, Guatemala; Pasmo Organization, Mariano Galvez University, Coatepeque, Quetzaltenango, Guatemala; Landivar University, Coatepeque, Quetzaltenango, Guatemala; Fundacion Para La Salud Integral de los guatemaltecos, Los Encuentros, Retalhuleu, Guatemala; Columbia University, new york, New York; Fundacion para la Salud Integral de Los Guatemaltecos, Fraijanes, Guatemala, Guatemala; Fundación para la Salud Integral de los Guatemaltecos, Burlington, VT; CU School of Medicine, Aurora, Colorado; University of Colorado School of Medicine, Aurora, CO, USA. ; Colorado School of Public Health, Aurora., Denver, Colorado

## Abstract

**Background:**

Professional development opportunities in research rural areas, low- and middle-income countries (LMICs) are scarce, nurses begin their careers with limited knowledge, training in research methods and trial implementation. Center for Human Development has conducted research in rural southwest Guatemala 2014. To address the need for qualified personnel in human subjects’ research, we developed a formal research training curriculum for nurses, aimed to prepare them for field work and a career in theoretical, practical research.

**Table 1:**
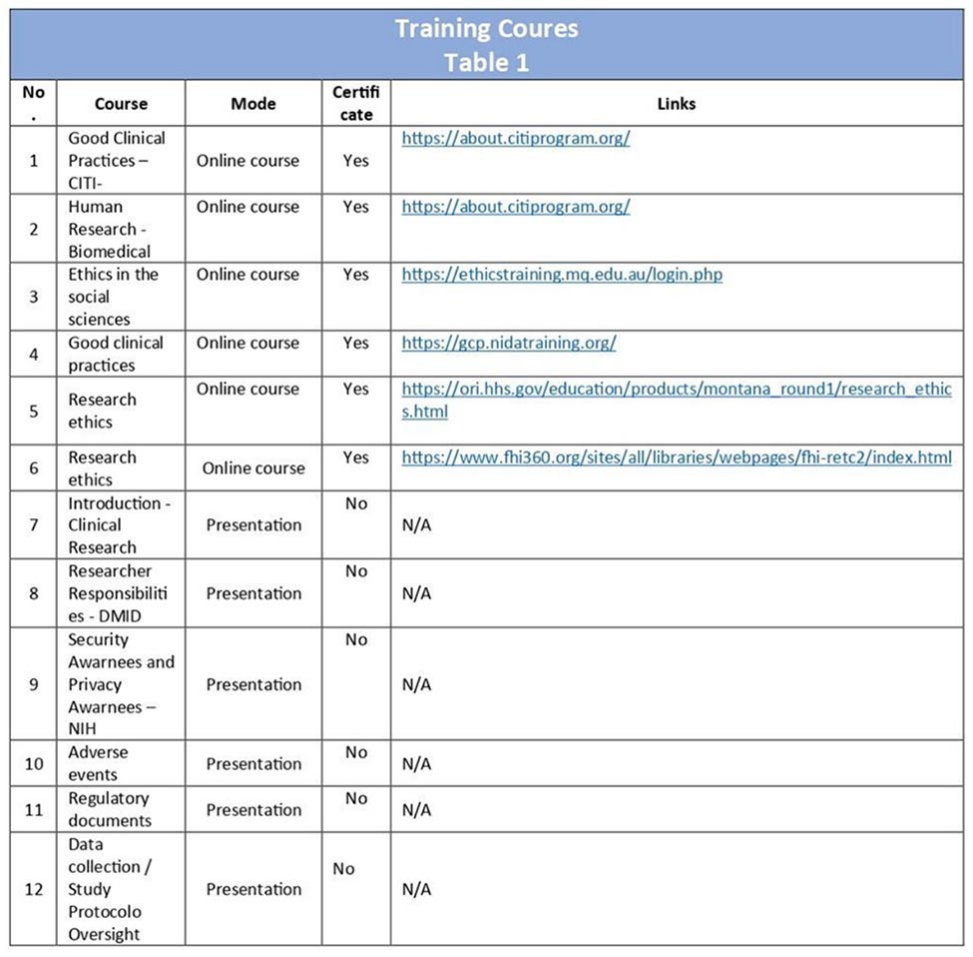
Training Coures

**Table 2:**
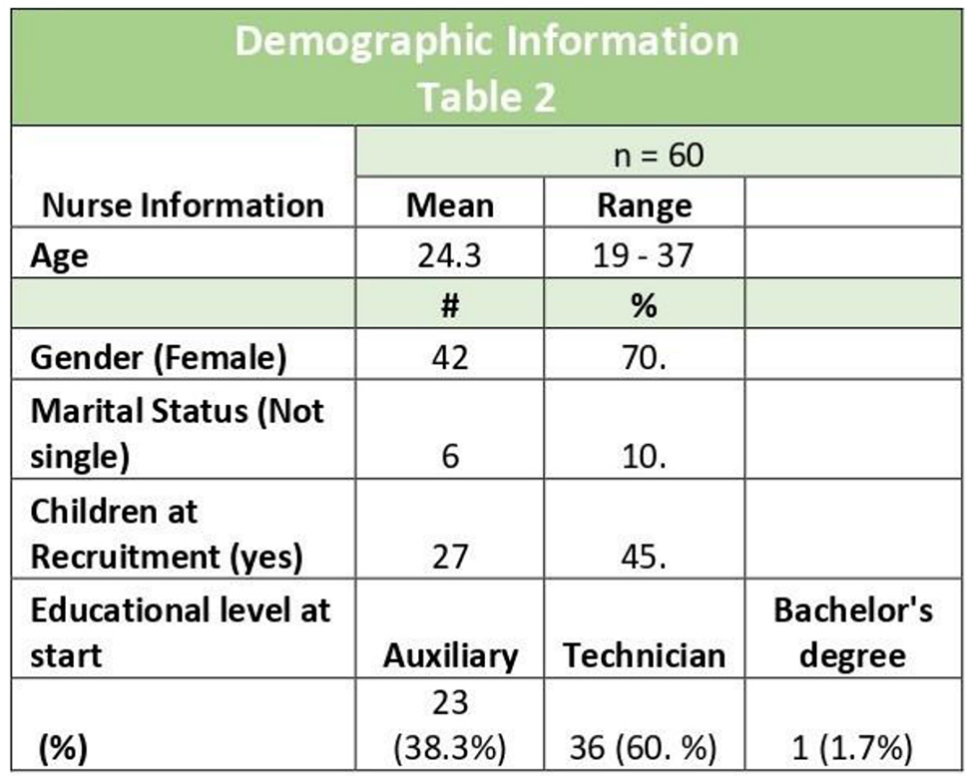
Demographic Information

**Methods:**

Following a needs assessment, the curriculum was designed using existing open-source certificate programs (i.e. Collaborative Institutional Training Initiative, Health and Human Services, included research ethics and Good Clinical Practice (GCP), via peer-to-peer internal presentations, reading assignments, practical sessions on protocol design, informed consent; sample collection, transport and storage; laboratory management; data quality, source documentation; REDcap Data and quality control. After four weeks of supervised fieldwork, nurses transitioned to independent work with decreased oversight.

**Results:**

From June 2015 to May 2024 60 nurses completed training program, including 42 (70.5%) women, ages 19-37 years; they included 23 (38.3%) auxiliary nurses, 36 (60.3 %) nurse technicians from the nearby Departments, Huehuetenango (n=1), Retalhuleu (n=4), Quetzaltenango (n=39), San Marcos (n=16). A subset or nurses (n=3) subsequently obtained a bachelor’s degree, advanced to a master’s, they are ethics committee reviewers, faculties at a local university. Of the 60 trained nurses, 11 continue to work at site; 3 currently are study coordinators.

**Conclusion:**

Conclusions: In conclusion, we have successfully developed and implemented a research nurse training curriculum in rural Guatemala, increasing capacity and providing an opportunity for professional development, especially impacting young women. Professional development training associated with opportunities to participate in research studies, contributed to increase retention, capacity and implement complex research studies in this rural area of Guatemala.

**Disclosures:**

Edwin J. Asturias, MD, Pfizer: Grant/Research Support

